# The science of adapted throws: a systematic search and narrative evidence synthesis

**DOI:** 10.3389/fspor.2025.1673489

**Published:** 2025-10-22

**Authors:** Exal Garcia-Carrillo, Cristian Alexis Lasso-Quilindo, Luz Marina Chalapud-Narváez, Antonio Castillo-Paredes, Claudio Farías-Valenzuela, Miguel Alarcón-Rivera, Rodrigo Yáñez-Sepúlveda, Lawrence W. Judge

**Affiliations:** ^1^ Department of Physical Activity Sciences, Universidad de Los Lagos, Osorno, Chile; ^2^ Department of Physical Activity Sciences, Faculty of Education Sciences, Universidad Católica del Maule, Talca, Chile; ^3^ Facultad de Ciencias Sociales y Humanidades, Corporación Universitaria Autónoma del Cauca, Popayán, Colombia; ^4^ Facultad de Ciencias Naturales, Exactas y de la Educación, Departamento de Educación Física, Recreación y Deporte, Universidad del Cauca, Popayán, Colombia; ^5^ Grupo AFySE, Investigación en Actividad Física y Salud Escolar, Escuela de Pedagogía en Educación Física, Facultad de Educación, Universidad de Las Américas, Santiago, Chile; ^6^ Escuela de Ciencias de la Actividad Física, el Deporte y la Salud, Universidad de Santiago de Chile (USACH), Santiago, Chile; ^7^ Escuela de Ciencias del Deporte y Actividad Física, Facultad de Salud, Universidad Santo Tomás, Talca, Chile; ^8^ Faculty of Education and Social Sciences, Universidad Andres Bello, Viña del Mar, Chile; ^9^ School of Medicine, Universidad Espíritu Santo, Samborondón, Ecuador; ^10^ Marieb College of Health and Human Services, Florida Gulf Coast University, Fort Myers, FL, United States

**Keywords:** para-athletes, paralympic athletes, sports for persons with disabilities, adaptive sports, track and field, biomechanical phenomena, sport, para sport

## Abstract

**Introduction:**

Paralympic throwing events have grown rapidly, yet the scientific evidence guiding technique, training and classification remains fragmented across biomechanics, physiology and psychosocial domains.

**Objective:**

To provide an evidence-based overview of adapted throws by comprehensively examining the peer-reviewed literature.

**Methods:**

A systematic search of PubMed, Scopus, and Web of Science (inception—Feb 2025) using the terms Paralympic OR adapted OR disability AND throw AND (shot OR discus OR javelin OR club). Inclusion criteria comprised: (i) Athletes with physical impairments who participate in Para Athletics throwing events, (ii) Non-disabled individuals studied in research explicitly designed to inform or understand Para Athletics throwing techniques. Data were synthesized narratively and clustered by study theme.

**Results:**

Nineteen studies (*n* = 345 para athletes; 14 sport classes) met the criteria. Biomechanical analyses identified release velocity (8.3–10.0 m·s^−1^ in F52–F55 shot put) and optimal angles (27.5°–37°) as key performance determinants. Assistive devices improved results by 8% in F32 athletes. Significant research gaps exist for visual impairment (F11–F13), intellectual impairment (F20), and prosthesis-user classes (F61–F64).

**Conclusion:**

While class-specific technical models are emerging, particularly for seated throwers, 38% of throwing classifications lack published research. Future studies should prioritize underrepresented classes and develop standardized assessment protocols.

## 1 Introduction

The capacity for high-velocity throwing emerged early in hominin evolution; morphological analyses show that the modern human hand combines precision grip with leverage-enhancing proportions that maximize projectile speed and accuracy (1). Comparative fossil work further suggests that such refinement constituted a primarily male adaptation that conferred advantages in inter-group combat and pursuit hunting (2). In contemporary sport these ancestral motor skills are formalized in the four throwing events of athletics: shot put, discus, javelin and (in Paralympic sport) club throw (3). Their disability-adapted version falls under Para Athletics. An adapted sport governed by World Para Athletics (WPA), the International Paralympic Committee (IPC) sanctioned discipline that mirrors Olympic track and field while accommodating physical, visual, and intellectual impairments through evidence-based classification rules (4).

Unlike their non-disabled counterparts, para throwers must generate competitive force output despite a wide spectrum of physical limitations ranging from reduced trunk control and limb deficiency to impaired coordination or sensory deficits (4). These diverse impairments, which are classified and grouped in WPA sport classes (e.g., F32–F57), necessitates biomechanical strategies that fundamentally differ from conventional throwing techniques, reflecting sport-specific adaptations to functional limitations (5, 6). To illustrate this, seated shot-put research shows that using an assistive pole results in higher release hand velocity (6.0 ± 1.5 m·s^−1^) when compared with throwing without a pole (5.3 ± 1.5 m·s^−1^), and that trunk and upper-body strength strongly correlate (*r* = 0.59–0.84) with throwing performance (7). Additionally, modifications in pole position or grip height influence trunk angular velocity and power, which directly affects shot displacement (8). Consequently, understanding performance in Para throws requires not only conventional sport-science tools, but also impairment- and equipment-specific biomechanical analyses that incorporate classification constraints (9).

Although performance determinants in able-bodied throwers have been examined extensively across biomechanics, neuromuscular physiology and training methodology (10), empirical coverage in Para throwing remains comparatively sparse (11, 12). Bibliometric mapping nevertheless indicates a recent upswing in publications, fueled by multidisciplinary collaborations that integrate engineering, medicine and social science to address the unique technical and psychosocial demands of athletes with disabilities (11, 13). These developments underscore a pressing need for a consolidated synthesis that translates fragmented findings into coherent, practice-ready guidance for coaches, classifiers and clinicians. Accordingly, this narrative review synthesizes and critically appraises the full spectrum of evidence on adapted throwing, with the dual aims of (i) distilling actionable, sport-class–specific performance principles for practitioners and (ii) delineating unresolved research questions that must be addressed to advance both competitive standards and athlete welfare. Therefore, the purpose of this study was to provide an evidence-based overview on adapted throws by comprehensively reviewing the available literature.

## 2 Methods

### 2.1 Sources of information

The protocol for this narrative synthesis followed structured recommendations outlined in the literature (14).

The literature search was conducted in the PubMed, Web of Science, and Scopus electronic databases using the following terms: “paralymp*” OR “disabled athlete” OR “para-athlet*” OR “para athlete*” OR “adaptive athlete*” OR “adapted athlet*” AND “throw*” OR “shot put” OR “discus” OR “javelin” OR “club throw”, without any date restriction, covering literature up to February 13, 2025. These databases were selected to provide comprehensive coverage of literature relevant to sports science and Paralympic research. It is important to note that the search strategy was designed to be inclusive, but it is possible that not every published study in this field was captured, as the yield is dependent on the indexing and accessibility of the consulted databases. Two authors (EGC & LWJ) independently screened titles and abstracts of all retrieved documents. Eligibility criteria comprised peer-reviewed articles examining athletes from all WPA throwing classifications (Table 1) participating in throwing events (i.e., shot-put, discus, javelin, club throw). This includes: (i) Athletes with physical impairments who participate in Para Athletics throwing events, (ii) non-disabled individuals studied in research explicitly designed to inform or understand Para Athletics throwing techniques (e.g., trunk rotation, force transfer, wheelchair propulsion in throwing, impact of specific impairments on technique). No language restrictions were applied to the search or selection process.

**Table 1:** Para athletics throwing event categories and athlete classifications.

Categories	Competing disabilities
10's	Para athletes with visual impairment (F11, F12, F13)
20's	Para athletes with intellectual impairment (F20)
30's	Para athletes with coordination impairments (i.e., hypertonia, ataxia, and athetosis) competing either seated (F31–F34) or standing (F35–F38)
40's	Para athletes with short stature (F40, F41)
	Para athletes with lower limb affectations (i.e., lower limb deficiency, leg length difference, impaired muscle power, or impaired passive ROM) competing without prosthesis (F42, F43, F44)
	Para athletes with upper limb affectations (i.e., limb deficiency, impaired muscle power, or impaired passive ROM) (F45, F46)
50's	Para athletes with limb deficiency, leg length difference, impaired muscle power, or impaired ROM competing seated (F51, F52, F53, F54, F55, F56, F57)
60's	Para athletes with limb deficiency or leg length difference competing with prosthesis (F61, F62, F63, F64)

ROM, range of movement.

Summary of classification criteria derived from World Para Athletics (16), table synthesized by the authors.

Exclusion criteria were applied when articles: (i) had topics not aligned with the focused purpose of the study; (ii) were published as notes, letters to the editor, books, dissertations, editorials, and rehabilitation-only studies. The same authors who conducted the study selection also performed the information extraction. Subsequently, studies were independently selected, and the results were deliberated to write the present narrative review. Furthermore, references from selected articles were scrutinized to identify additional relevant papers.

**Figure 1:**
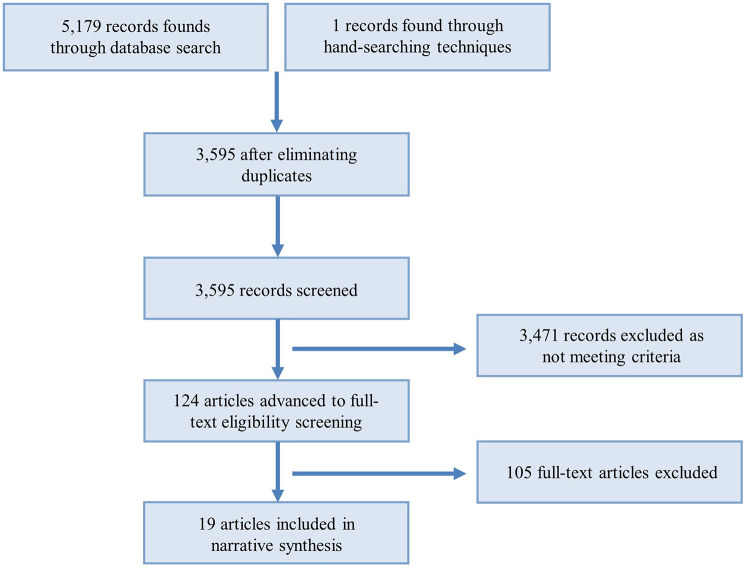
Study selection flowchart.

Following the Preferred Reporting Items for Systematic Reviews and Meta-Analyses (PRISMA) framework (15), we systematically reviewed the literature, with the screening process visualized in Figure 1.

## 3 Results

### 3.1 Literature search

A total of 5,179 publications were initially identified through the literature search. Following title and abstract screening, 124 articles were deemed potentially eligible for inclusion. After duplicate screening, removal of qualitative studies, and a supplementary manual review of reference lists, 19 studies ultimately qualified for quantitative synthesis (refer to Figure 1 for the study selection flowchart).

### 3.2 Study design of included studies

The methodological designs of the studies included consisted of one quasi-experimental study (17), eight cross-sectional studies (18–24), four case studies (8, 18, 25, 26) primarily detailing world-class throwers' technical parameters, and two retrospective studies (27, 28). One study is a validation and reliability study (29). Finally, one study is a literature review (30).

### 3.3 Description of included studies

Table 2 summarizes the key characteristics and primary findings of the 19 studies meeting inclusion criteria (7, 8, 17–33), yielding a pooled sample of 345 athletes across 14 WPA sport classes (F32–F58 seated throwers; F61–F63 limb-deficient). One of the included studies involved individuals without disabilities (29). Shot-put was the most examined event [42.1% (8, 19, 22, 25–27, 31, 32)], followed by discus [15.8% (18, 20, 21)], 10.5% of studies assessed multiple events (e.g., shot put, javelin, etc.) (17, 28), 15.8% of studies did not specify the throwing event (23, 24, 33), no studies addressed club throw.

**Table 2:** Key characteristics and primary findings of studies on para athletics throwing.

Authors (year)	Study design (category)	Sport-class participants	Outcome & magnitude (key variables)	Practical application/coaching take-away	Evidence level*
Alhumaid et al. (18)	Case study (biomechanics)	F33 male discus WR holder (*n* = 1)	Release vel. = 16.77 m·s^−1^; ∡ = 37°; accel-path = 1.85 m; launch-h = 1.36 m	Lengthen acceleration path via trunk rotation drills; cue release angles 35–38°	4
Chow et al. (19)	Cross-sectional (kinematics)	Seated shot-putters (*n* = 17)	Vel. 5.3–7.8 m·s^−1^ (all classes); ∡ 21.2–34.4°; ROM/shoulder speed ↑ with class & distance (*p* < 0.05)	Focus on training upper arm angular speed; balance release angle/height; develop shoulder girdle ROM	2B
Frossard (27)	Retrospective (biomechanics)	Seated shot-putters F32–F34, F52–F58 (*n* = 68/46)	Perf. dispersion: + 1.46 m (M F54 → F58); +1.06 m (F F55 → F58); Linear class-perf. trend (outliers F33/F52)	Use comparative matrices for evidence-based classification; explore performance continuum models for talent identification	2B
Frossard et al. (31)	Observational (kinematics)	Gold medalists F52–F55 shot (*n* = 8/3)	Vel. ↑ 8.30 → 9.96 m/s (M), 4.58 → 8.50 m/s (F); ∡ ↑ 27.5 → 32.5° (M), 9.0 → 34.5° (F)	Use release vel./∡ as key performance metrics; Gender-specific benchmarks for training	2B
Frossard et al. (20)	Descriptive (biomechanics)	F33–F34 seated discus (*n* = 12)	Configurations: 3–6 contact pts (25–42% users); 58% standing throwers; 50% used straddle seats	Test varied contact points (3–6) and seating setups (straddle/stool/chair) in throwing frame	4
Frossard et al. (21)	Descriptive (biomechanics)	F33–F34 seated discus (*n* = 12)	4/30 feet metrics *r* > 0.5: ↔/↕ spacing (F34), back foot position/↔ spacing (F33)	Optimize ↔/↕ foot spacing in frame design; Prioritize back foot positioning in F33	4
Garcia-Carrillo et al. (25)	Case study (load monitoring)	F54 female shot WR (*n* = 1)	2-wk taper ITL ↓37.9%; Performance ↑7.6%; No Δ in wellbeing	Implement 2-week tapers for F54 throwers; Monitor ITL via s-RPE despite wellbeing	4
Garcia-Carrillo et al. (22)	Cross-sectional (anthropometrics)	Male shot-put F56/F42/F63 (*n* = 5)	Somatotype: endo-mesomorph (4.4–6.9–1.0); fat mass 25.7 ± 2.8%; handgrip 66.4 ± 6.7 kg (6.5% asymmetry)	Shot-putters exhibit high muscularity and body fat; monitor handgrip asymmetry for potential performance implications	2B
Garcia-Carrillo et al. (28)	Retrospective (epidemiology)	Mixed class/event (*n* = 42/18)	12-month injury prevalence: 40% (95% CI: 27.5–53.4); shoulder (22.8%) & elbow (25.3%) most affected; strains (30%), tendinopathies (25%); 88.6% training-related	Implement sport-specific injury prevention programs focusing on upper extremities (shoulders/elbows), especially for male	2B
Holdback et al. (30)	Narrative review (biomechanics)	Classes F33–F55	Pole use ↑ performance in F32 (*p* < 0.05); significant class effect (*p* < 0.001); high individual variability within classes	Throwing pole may benefit F32 athletes; individual equipment optimization needed across classes	5
Holdback et al. (26)	Comparative (biomechanics)	F34 seated shot (*n* = 1)	Pole resonance 5–6 Hz; deflection method valid vs. direct measurement	Non-instrumented pole force measurement feasible; resonance effects warrant further study	4
Holdback et al. (8)	Case study (kinetics)	F34 seated shot (*n* = 1)	Lower grip (−75 mm) ↑ trunk vel. (64% contribution) & power; performance improvement after 4 sessions	Individualized pole grip optimization (lower position) improves trunk biomechanics and performance	4
Hyde et al. (7)	Comparative (biomechanics)	SCI athletes F51–F54 (*n* = 8/2)	Pole ↑ hand speed (6.0 vs. 5.3 m·s^−1^, *p* = 0.02); grip (*r* = 0.59–0.77) & trunk strength (*r* = 0.50–0.84) correlate with performance	Strength tests should inform classification; consider separate pole/no-pole competitions	2B
Lee et al. (32)	Cross-sectional (biomechanics)	F53–F58 shot (*n* = 16)	Release speed best predictor (*r* = .95, *p* < .01); speed-angle correlation (*r* = 0.37 combined, *r* = 0.57 women)	Prioritize release-speed drills; angle consistency secondary; gender-specific approaches may be needed	2B
Mahmoudkhani et al. (33)	Cross-sectional (isokinetics)	F55–F57 male (*n* = 21)	Intl. > Nat. shoulder IR & elbow flexor torque (14%–18%)	Prioritize balanced shoulder/elbow strengthening; use findings for talent development	IIb
Singh et al. (23)	Cross-sectional (psychology)	Standing and seated throwers (*n* = 9)	No sig. differences (*p* > 0.05) in mental toughness: motivation, confidence, energy control, attention, visualization, attitude control	Mental toughness profiles were similar across groups; this reinforces psychological training as core regardless of throwing position	2B
Singh et al. (24)	Cross-sectional (psychology)	Standing and seated throwers (*n* = 16/4)	No sig. differences in physical, verbal aggression or hostility; anger significantly ↑ in seated group (*p* < 0.05)	Prioritize anger regulation in seated throwers; include psychological screening & pre-competition support	2B
Spathis et al. (29)	Cross-sectional (talent identification)	non-disabled physically active (*n* = 13/15)	High test-retest reliability (mean ICC = 0.89); strong correlations with criterion throws (mean rs = 0.76); seated: force/target throw; standing: broad jump	Use seated force/target throw and broad jump to ID throwing potential; screen by throwing type (linear, rotational, overhead)	2B
Voroshin et al. (17)	Quasi-experimental (training)	F54, F57, F32 and F33 shot-put, discus, and javelin throwers (*n* = 5/1)	Mean improvements: shot +3.15 ± 0.69 m (*p* < 0.05), discus +0.85 ± 0.13 m (*p* < 0.01), javelin +2.53 ± 0.41 m (*p* < 0.001); 10 PBs, 11 medals (8 gold)	Use 3D video modeling for biomechanical tracking and targeted corrections; apply ≥3 technique sessions/week in pre-comp period	2B

cat, sport category; ITL, internal training load; *n*, number of participants; perf, performance; s-RPE, session-Rating of Perceived Exertion; vel, velocity; *X*/*Y*, indicates a total of *X* male and *Y* female participants; ∡, angle; ↔, medio-lateral; ↕, antero-posterior; ↑, increase; ↓, decrease; *Δ*, change.

* Evidence levels follow Oxford Centre for Evidence-Based Medicine (I = RCT → V = expert opinion) (34).

### 3.4 Study characteristics

Of the 19 studies included, biomechanical analyses (18, 20, 21, 26, 27, 30) represented 36.8% (*n* = 7), while kinematics (19, 31), kinetics (8), and isokinetics (33) were each examined in one study (5.3% each). Remaining studies explored load monitoring (25), anthropometrics (22), injury epidemiology (28), psychological factors (23, 24), talent identification (29), and training methods (17), alongside one narrative review analyzing equipment trends and performance impacts of throwing poles in seated shot-put across Paralympic classifications (30). Biomechanical research predominantly examined sport-specific movement patterns, while other categories provided complementary performance insights.

## 4 Discussion

The purpose of this study was to provide an evidence-based overview of adapted throws through a comprehensive review of the available literature. It was found that most of studies conducted on adapted throwing were cross-sectional, including male and female athletes, with only one investigation with a quasi-experimental design (17). This scarcity of longitudinal or intervention studies highlights a critical gap in understanding how training adaptations, biomechanics, and athlete-specific factors (e.g., impairment type, classification, or anthropometrics) influence performance over time.

### 4.1 Biomechanics/kinetics/kinematics/isokinetics

Studies identified the use of biomechanical analysis in kinematic and kinetic characteristics during training and international competitions. In addition, the use of training methods with biomechanical data that contribute to the optimization of physical performance for performance improvement in field events. This information provides relevant data for classification based on scientific data. Release velocity and release angle remain the two most powerful predictors of seated-shot and seated-discus distance (32). Among elite F52–F55 shot-putters, release speed increases from 8.3 m·s^−1^ to 10.0 m·s^−1^ across successive sport classes, paralleled by a systematic rise in release angle from ≈27.5° to 32.5° (32). Detailed three-dimensional analyses of gold-medal F33 discus throwers reveal that a 1.85 m implement-acceleration path completed in 0.40 s can generate tangential kinetic energy >9 J and a release speed of 16.8 m·s^−1^ at 37° (18). Two seated-discus studies further demonstrate that whole-body trunk orientation and foot-block placement on the throwing frame each account for >30% of the variance in performance (20), underscoring the necessity of sport-class–specific technical models (21).

Recent single-subject evidence offers validation of these mechanistic principles tracking a world-class non-disabled shot-putter through a nine-month Olympic-qualification macrocycle, combining weekly 300 Hz motion-capture and radar-based (Trackman) profiling with a tightly periodized special-strength block. The intervention—loaded (10 kg wt. vest) kneeling-shot throws, lying medicine-ball drop push drills, concentric-only bench press at 0.8 m·s^−1^, and resisted overhead half-turns yielded a 1.40 m competition improvement (17.83 → 19.24 m; +7.9%). Mean release angle fell from 42.1° to 36.9°, while peak release velocity increased from 12.4 to 13.0 m·s^−1^, demonstrating that targeted strength drills can restructure kinematic sequencing rather than merely augment force magnitude (35). Because seated F52–F58 throwers operate under analogous constraints: minimal lower-limb impulse and heavy reliance on trunk–shoulder rotational kinetics, velocity-centered, angle-attenuating prescriptions of this type appear directly transferable, provided they are adapted to classification-specific stability regulations.

Assistive-device research quantifies the mechanical contribution of poles and chair configurations. Tokyo-2020 analytics demonstrated an ∼8% distance gain in F32 athletes when a pole was used vs. no-pole conditions (30). Force-deflection testing later showed peak pole loads exceeding 450 N, with lower grip heights increasing trunk-generated power by ≈10% across four training sessions (26). A crossover trial in spinal-cord-injured throwers corroborated that pole use elevates hand speed at release independent of seat configuration (7). At the muscular level, international F55–F57 athletes exhibit 14%–18% greater concentric internal-rotator and elbow-flexor torque than national peers, highlighting the importance of targeted isokinetic work (33).

### 4.2 Anthropometrics

While anthropometrics in able-bodied throwers has some evidence suggesting a relationship between body height, mass, and performance (36), the influence of these factors in Paralympic throwers remains less explored. Current research suggests that particular anthropometric characteristics can positively influence physical performance in para athletes from various sport classes (37). However, body composition assessment in athletes with disabilities faces some challenges, primarily due to the absence of standardized methodologies for this population (38). Highly-trained shot-putters have shown a meso-endomorphic profile (sum of skinfolds ≈ 73 mm; stature ≈ 1.83 m) with small bilateral grip-strength asymmetries (22). Further studies are needed in this field to establish athlete profiles in different classes and events, which would be useful for talent identification and performance optimization. Talent-identification testing shows strong correlations (*r* > 0.70) between Seated Force Throw/Target Throw scores and eventual seated-event performance, whereas the Standing Broad Jump best predicts ambulant throw success (29). These findings can guide early classification and training decisions.

### 4.3 Psychology

Only one controlled investigation has profiled psychological traits in world-class throwers, reporting equivalent mental-toughness scores (MTQ-48 total) but markedly higher anger and hostility on the POMS in seated vs. standing Paralympians (Cohen's *d* ≈ 1.2 for anger) (23, 24). Such dysregulated affect threatens attentional control at release and increases injury-risk behaviors, indicating a clear need for integrated mental-skills and psychosocial support. Judge and colleagues advance this agenda by articulating a sport-social-work framework in which licensed social workers deliver emotion-regulation training, navigate classification-appeal stressors and coordinate community-based resources, functions that reduced self-reported distress 17% in a pilot F55–F57 cohort (39). From the coaching side, transformational leadership in disability sport is driven primarily by mastery experiences (*β* = 0.41, *p* < 0.001) and peer persuasion (*β* = 0.32, *p* < 0.01), whereas formal accreditation shows no independent effect; these findings derive from a multivariate analysis of 206 Para Athletics coaches in which the predictive model explained 48% of variance in athlete-rated leadership behaviors (40). Collectively, the evidence argues for dual-channel interventions: sport-social-work support for athletes and mentorship-based efficacy building for coaches, to optimize both performance and psychosocial welfare in Paralympic throwing.

### 4.4 Training and performance optimization

A six-month, quasi-experimental program that paired weekly 3-D motion capture with coach-mediated feedback elicited statistically meaningful personal bests of 1.4–2.6 m in F32, F33, F54 and F57 throwers, confirming the value of real-time kinematic auditing and iterative cueing for technical refinement (17). Acute potentiation protocols convey an additional competitive edge: a single warm-up sequence comprising 45–60 medicine-ball contacts elevated release velocity by 2%–4% in F44 athletes, an effect comparable to post-activation performance enhancement observed in non-disabled cohorts (41). In a world-champion F54 shot-putter, a two-week taper that reduced external load by 40% but preserved velocity-specific drills generated a season best and a 7.6% increase in throwing distance, illustrating the primacy of movement velocity during unloading phases (25). Resistance training prescriptions targeting the internal shoulder rotators and elbow flexors directly address the 14%–18% torque deficits documented in national-standard vs. international F55–F57 athletes (33). Injury-related studies further recommend coupling these upper-limb stimuli with trunk-stability and core-bracing drills (28, 42, 43).

Five investigations describe the 30-series (F31–F34) cohort (18, 20, 21, 27, 30). Competition distances in elite male F33 athletes span 22.17–34.65 m and in F34 athletes 17.41–32.42 m (18, 20), with optimal release angles converging on 35–37°, echoing able-bodied norms (19). Peak implement velocity of 16.77 m·s^−1^ has been recorded in F33, whereas F34 data remain absent. Cane utilization improves performance ∼8% in F32 throwers, probably via augmented trunk-flexion impulse, and pole-deflection testing provides a practical surrogate for quantifying this workload (27). A simple force-deflection method, attaching a linear-potentiometer to the pole, allows coaches to quantify the impulse applied and ensure progressive overload without exceeding stability limits (27). Grip height is equally consequential: lowering the pole by 5 cm increased trunk power 10% in a controlled trial (44), corroborating syntheses linking grip mechanics, trunk-flexion force and distance (45). These findings endorse pole-height prescriptions and somatotype considerations as integral components of class-specific technical models (22).

Evidence for the 50-series (F51–F57) is more extensive (7, 22, 25, 27, 30–33). Release velocity rises systematically with both classification and performance tier—8.30 → 9.96 m·s^−1^ in males and 4.58 → 8.50 m·s^−1^ in females—accompanied by parallel increases in release angle (males 27.54° → 32.47°, females 9.02° → 34.52°). Load–performance monitoring during taper phases reinforces concentric bar speed (not absolute tonnage) as the principal predictor of competitive outcome. Isometric mid-thigh-pull and seated chest-press peak force correlate strongly with throw distance (*r* ≥ 0.72), offering objective benchmarks for athletes with limited lower-limb function (45). Resistance training narrows the upper-limb strength gap while simultaneously reducing shoulder-girdle injury incidence (46, 47), which is critical for ambulant throwers (e.g., F42, F63) characterized by high fat-free mass and attendant joint stress (22).

Integrating velocity-specific resistance work, trunk-stability conditioning and external-load monitoring yields a mechanistically coherent strategy for maximizing performance while mitigating overuse risk. Nevertheless, empirical gaps persist in classes F11–F13, F20, F35–F38, F40–F41, F43–F46, F52 and F61–F64, highlighting the need for multi-center motion-capture cohorts and randomized training trials to extend these evidence-based prescriptions across the full spectrum of Paralympic throwing.

### 4.5 Evidence gaps

Despite a sustained increase in Para Athletics research (11), critical gaps persist in throwing events literature. Longitudinal or interventional data remain scarce for visual-impairment (F11–F13), intellectual-impairment (F20) and several limb-deficient and coordination-impairment classes (F35–F38, F40–F41, F61–F64), underscoring the need for evidence in these under-represented groups (48). There is a need for longitudinal data and randomized controlled trials in these groups to establish evidence-based practices and more utilization of technology. For example, trials comparing velocity-based and percentage-based loading schemes, alongside integrated biomechanical–psychological interventions, would clarify causal pathways between training stimuli, technical execution and competitive outcomes. By uniting precise kinetic targets with comprehensive psychosocial support, researchers and practitioners can transition from descriptive analytics to predictive, personalized performance models, advancing both competitive standards and athlete health across the full spectrum of adapted throwing. Further, intervention designs combining biomechanical feedback (e.g., kinetic targets via velocity-based training) with psychological strategies (e.g., imagery, self-talk) could reveal causal mechanisms linking training stimuli, technical execution, performance outcomes, and athlete wellbeing. By extending models proven effective in non-disabled populations to Para Athletics, researchers can move beyond descriptive analytics to develop predictive, personalized performance systems, ultimately elevating competitive standards and long-term health across the Paralympic throwing spectrum.

### 4.6 Limitations and strengths

Available literature demonstrates significant methodological limitations and outcome variability, making meta-analysis nonviable. This evidence synthesis presents several limitations that should be noted. The predominance of cross-sectional designs within the included studies restricts causal conclusions regarding training efficacy, while inconsistent methodological approaches to biomechanical data collection limit direct comparability across studies. Furthermore, the scarcity of research involving visual impairment classes (F11–F13) and athletes competing with prostheses (F61–F64) limits generalizability to these populations. Despite these limitations, this synthesis offers substantive contributions through its systematic adherence to PRISMA guidelines, its exclusive focus on competition-relevant performance metrics from elite throwers, and its novel integration of kinematic, kinetic, and training load data across the full spectrum of seated and ambulant sport classes.

### 4.7 Future directions

Next steps in the field should aim to develop longitudinal intervention studies employing randomized designs to explore training effects in underrepresented sport classes (e.g., athletes with visual impairment, athletes competing with prosthesis). The development of standardized biomechanical protocols with high-frequency motion capture would enable robust cross-study comparisons and the establishment of class-specific technical models. Technological innovations such as wearable inertial sensors could bridge the gap between laboratory and field-based monitoring of release parameters. Given the heterogeneity of impairments in Para athletics, future research should examine how anthropometrics interact with classification-specific biomechanics. Simultaneously, there is a need to investigate psychosocial variables (e.g., emotion regulation among seated throwers), given its strong associations with mental toughness and athletic resilience in disabled athletes. Achieving these aims will require coordinated collaboration among researchers, classifiers, and Paralympic organizations to advance both empirical knowledge and practical applications in Para Athletics.

## 5 Conclusion

Adapted throws research ranged from biomechanical analyses to descriptive case reports. This review emphasizes the scant but expanding body of evidence on adapted throwing. Studies report ideal seated shot-put release-angles around 35–37° and discus acceleration pathways nearing 1.8 m, release velocity and angle continue to explain the greatest portion of performance difference across seated and ambulant sport classes. Equally significant is the increasing understanding whether the configuration of assistive devices, specifically pole use, grip height, and throwing chair geometry, can modulate trunk-generated power by nearly 10%. This underscores the necessity of specific sport-class equipment guidelines that strike a balance between maximum performance potential and competitive equity. Key findings highlight the efficacy of trunk-stability conditioning, and assistive-device optimization (e.g., pole mechanics in F32 throwers) for performance enhancement. Psychological and coaching interventions also demonstrate promise, particularly in addressing emotion regulation and injury-risk behaviors in seated athletes. Nevertheless, the field remains limited by a predominance of cross-sectional studies, inconsistent methodologies, and the absent of longitudinal or randomized controlled trials.

No published studies were found for athletes in classes F11–F13, F20, F35–F38, F40–F41, F43–F46, F52, or F61–F64, despite the growing interest in Para Athletics research, representing a critical gap in the literature. The development of Paralympic throwing as an evidence-based discipline requires collaborative efforts across research, classification, and coaching domains. By addressing current limitations and expanding investigations in underrepresented groups, the field can advance toward more personalized and equitable training approaches while raising competitive standards across all classifications.

## Data Availability

The original contributions presented in the study are included in the Article/Supplementary Material, further inquiries can be directed to the corresponding author.
